# Progress in the Development of Chitosan-Based Biomaterials for Tissue Engineering and Regenerative Medicine

**DOI:** 10.3390/biom9090470

**Published:** 2019-09-10

**Authors:** Bolat Sultankulov, Dmitriy Berillo, Karina Sultankulova, Tursonjan Tokay, Arman Saparov

**Affiliations:** 1 Department of Chemical Engineering, School of Engineering, Nazarbayev University, Nur-Sultan 010000, Kazakhstan; 2 Water Technology Center (WATEC) Department of Bioscience - Microbiology, Aarhus University, Aarhus 8000, Denmark; 3 Department of Biotechnology, Al-Farabi Kazakh National University, Almaty 050040, Kazakhstan; 4 Karaganda Medical University, Karaganda 100000, Kazakhstan; 5 School of Science and Technology, Nazarbayev University, Nur-Sultan 010000, Kazakhstan; 6 School of Medicine, Nazarbayev University, Nur-Sultan 010000, Kazakhstan

**Keywords:** chitosan, biomaterials, tissue engineering, regenerative medicine, bone, cartilage

## Abstract

Over the last few decades, chitosan has become a good candidate for tissue engineering applications. Derived from chitin, chitosan is a unique natural polysaccharide with outstanding properties in line with excellent biodegradability, biocompatibility, and antimicrobial activity. Due to the presence of free amine groups in its backbone chain, chitosan could be further chemically modified to possess additional functional properties useful for the development of different biomaterials in regenerative medicine. In the current review, we will highlight the progress made in the development of chitosan-containing bioscaffolds, such as gels, sponges, films, and fibers, and their possible applications in tissue repair and regeneration, as well as the use of chitosan as a component for drug delivery applications.

## 1. Introduction

Development of biomaterials is an active research field with the purpose of designing scaffolds for the regeneration of tissues and organs damaged by disease or injuries. Defining and designing appropriate material for tissue engineering is a critical step in tissue engineering and regenerative medicine [1]. In the past few decades, significant attention has been given to natural polymers because of their biocompatibility and structural similarity to the extracellular matrix components. Abundant availability and unique biological activity of each natural polymer makes them a matching candidate for the development of novel natural or/and semi-synthetic materials closely resembling the natural structure and functionality of tissues required for successful regeneration. Starch, collagen, alginate, cellulose, hyaluronic acid, chitin, and chitosan (CS), are attractive natural polymers suitable for tissue regeneration. CS is a linear natural carbohydrate biopolymer derived from chitin with a structural similarity to glycosaminoglycans of the extracellular matrix (ECM) implicated in cell–cell adhesion [2]. The hydrophilic structure of CS promotes cell adhesion, proliferation, and differentiation of different types of cells and the polycationic nature of CS at a mildly acidic condition allows immobilization of negatively charged enzymes, proteins, and DNA for gene delivery [3,4]. CS for tissue engineering and regenerative medicine could be designed in various forms, such as hydrogels, sponges, fibers, sheets, films, and other structures [5].

## 2. Structure and Physico-Chemical Properties

**Figure 1:**
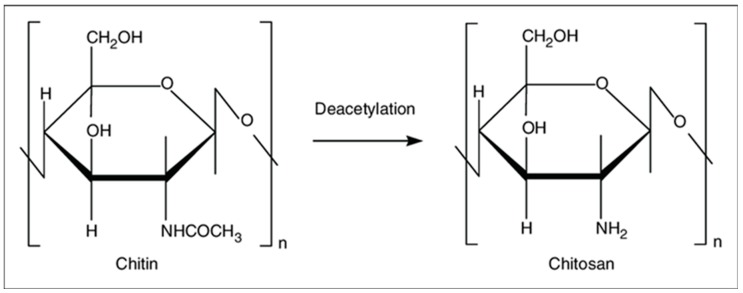
Chitin and chitosan structure.

Chitin is the second most abundant natural polymer [6] and consists of 2-acetamido-2-deoxy-β-d-glucose through a β (1→4) linkage and is extracted from the shells of marine crustaceans, insects, or fungi. Chitin is insoluble in water and most organic solvents, and therefore its use in biomaterials fabrication is limited. CS is a linear polysaccharide derived from partial deacetylation of chitin, as shown in Figure 1. It is a copolymer of randomly located (1→4)-2-acetamido-2-deoxy-β-d-glucan (*N*-acetyl d-glucosamine) and (1→4)-2-amino-2-deoxy-β-d-glucan (d-glucosamine) units. The number of amino groups as a ratio between d-glucosamine to the sum of d-glucosamine and *N*-acetyl d-glucosamine is indicated as a deacetylation degree (DD) and should be at least 60% for CS. The deacetylation of chitin is conducted by chemical hydrolysis (alkaline conditions) [7] or by enzymatic hydrolysis (chitin deacetylase) [8]. CS is soluble in dilute organic acids such as acetic acid [9], as well as diluted hydrochloric acid, and further modification of CS is accessible due to the availability of amino groups [6]. The fungal source of CS is preferred at the industrial scale because of its narrower molecular mass distribution, all-year-round availability, more controlled and scalable production, and less immunogenicity in comparison to a seafood source, which could cause allergies and limit biomedical application [7].

The physical properties of CS depend on several factors, such as the molecular weight, DD, and purity of the product [10]. CS solubility is pH dependent [11] and it is soluble in diluted acids achieved by protonation of the amino groups of the d-glucosamine residues [12]. Availability of protonated amino groups enables CS to form complexes with metal ions [13,14], natural or synthetic anionic (poly(acrylic acid)) polymers [15], lipids, proteins, and DNA. CS-based scaffolds can be chemically cross-linked by glutaraldehyde, oxidized dextran or other oxidized carbohydrates, 1,1,3,3-tetramethoxypropan, and genipin [15,16,17]. It is important to note that CS is a unique semi-natural positively charged polysaccharide at acidic conditions [18]. This property is used to develop CS-based polyelectrolytes for the preparation of films via a layer-by-layer deposition technique [15]. The amino groups of CS could react with aldehyde groups through reductive amination [9]. Hydroxyl groups along a CS chain enables etherification and esterification [19]. In addition, CS possesses important properties, such as high biocompatibility, biodegradability, antibacterial activity, non-antigenicity, and high adsorption properties that make CS a good candidate for tissue engineering and other biomedical applications [8].

## 3. Chitosan in Tissue Engineering and Regenerative Medicine

### 3.1. Chitosan for Wound Healing

Skin regeneration is a complex process that consists of four overlapping phases—hemostasis, inflammation, proliferation, and tissue remodeling [20]. In other words, skin regeneration is a dynamic process involving blood elements, extracellular components, soluble factors, and cells [21]. Therefore, the treatment of skin lesions requires dressing that not only ensures physical protection of the wound but also enhances the healing, provides antimicrobial protection, and reduces scar formation [22].

CS has very strong hemostatic activity which is not dependent on host coagulation pathway [23] but depends on CS’s molecular weight and DD [24,25]. The number of amine groups has a direct effect on blood coagulation, where moderate DD (68.36%) causes the formation of a mesh-like structure within CS, thus facilitating interaction with blood components, whereas higher DD results in stronger hydrogen bonds within CS causing the formation of a crystalline structure with limited ability to interact with red blood cells [24,25,26,27]. Higher molecular weight could further increase the procoagulation effect due to higher interaction between polyelectrolytes [28,29]. There are several CS containing hemostatic products available on the market and approved by the Food and Drug Administration of the United States (FDA), such as Celox^®^, HemCon^®^, Axiostat^®^, Chitoflex^®^, and Chitoseal^®^ [30].

In addition to the hemostatic effect of CS, it was shown that CS affects all stages of healing in various ways. It was shown that CS induces migration of neutrophils [31], and neutrophil-like HL60 cells secrete IL-8, a potent neutrophil chemokine, in response to CS in direct correlation with the level of *N*-acetylation [32]. CS has an immunomodulatory effect which is important for the wound healing process and depends on DD [33]. It was shown that micro- and nano-sized CS particles induce inflammasome formation by macrophages [33,34,35,36]. In contrast, macro-sized CS scaffolds inhibit the release of IL-1β and thus the formation of inflammasomes in mouse and human macrophages in vitro [37], making the use of macro-sized CS scaffolds rational when excessive inflammation is present. Moreover, CS also affects the expression of growth factors by increasing TGF-β1 expression in the early post-injury phase [38] and decreasing it in later stages by binding to anionic growth factors [39]. High DD CS stimulates proliferation of dermal fibroblasts allowing fibrous tissue formation and re-epithelialization [40,41]. The polyelectrolyte complex-based cryogel of CS-gelatin-oxidized dextran (Ox.D) and different CS-oxidized dextran compositions showed elastic modulus in the range 2.7–14.3 + 0.4 kPa. The proliferation rate for cell culture of fibroblasts on CS-Ox.D-gelatin (1:1:1) increased significantly compared to the other CS compositions with Ox.D due to internal porosity of pore walls [15,16]. CS containing scaffolds for wound healing could be made as 2D (films and fibers) and 3D (gels and sponges) with the properties required for wound management [42]. The antimicrobial effect of CS could be enhanced by the addition of antimicrobial agents. In a recent study, a complex CS-cordycepin hydrogel with increased antimicrobial activity was developed without the addition of any cross-linking agents via a freeze-drying method where negatively charged cordycepin adhered to positively charged CS chains [43]. In another study, textile polyethylene terephthalate composed of layer-by-layer coated CS was loaded with chlorhexidine and the mechanical stability of the composite was increased by thermal post-treatment which also increased the duration of chlorhexidine release up to 7 weeks [44]. CS alone or in complex with other natural polymers is also used as a part of asymmetric membranes, usually in an underlying layer that is in contact with the damaged skin [45]. Addition of nanoparticles (NPs) into hydrogels is another strategy used in biomaterial preparation [13]. Shah and colleagues developed triple-component nanocomposite film that contained CS-silver-sericin and was loaded with moxifloxacin. The obtained films possess not only high antimicrobial activity against methicillin-resistant *Staphylococcus aureus* (MRSA) strains (clinical isolates) but also support wound healing in a rat model, similar to commercial wound dressings [46]. Most of the CS composite films containing collagen have intrinsic properties to induce healing, but the drawback is an allergic reaction to non-human collagen and therefore other safe substitutes are in demand. For example, human keratin-CS membrane with improved mechanical properties produced by the UV-crosslinking method shows potential as a wound dressing [47]. CS-chondroitin sulfate-based polyelectrolyte complex shows an efficient antimicrobial effect and cytocompatibility suitable for wound healing applications [48]. Furthermore, positively charged CS containing biomaterials could be loaded with growth factors and cytokines to improve their performance in the wound healing process. In a recent study, CS NPs prepared through ionotropic gelation with tripolyphosphate [49] were loaded with granulocyte-macrophage colony-stimulating factor (GM-CSF) as a part of a nanocrystalline cellulose–hyaluronic acid composite prepared by a freeze-drying method [50]. Loading efficiency of GM-CSF was as high as 97.4 ± 1.68% with sustained release of ~100% over 48 h and in vivo experiments have shown that composites loaded with encapsulated GM-CSF in CS NPs induce greater wound closure compared to the composite alone [50]. Polycaprolactone nanofibers loaded with CS NPs containing GM-CSF also showed accelerated wound closure [51]. Modification of CS with peptides also promotes wound closure, for example, CS hydrogels made from Ser-Ile-Lys-Val-Ala-Val-chitosan macromers [52] when applied in vivo induces collagen expression, angiogenesis, expression of TGF-β1, and inhibits the expression of TNF-α, IL-1β, and IL-6 mRNA in a mouse skin wound model [53]. CS could be further modified to increase affinity for the growth factors. For example, developed heparin-like polysaccharide (2-*N*, 6-*O*-sulfated CS) has a high affinity to the vascular endothelial growth factor in comparison to heparin due its higher sulfonation degree [54,55].

### 3.2. Bone and Cartilage Regeneration

During the development of biomaterials for bone and cartilage regeneration, it is necessary to not only create a scaffold that is biocompatible and biodegradable, but also contains suitable mechanical properties with interconnected pores [15] that supports the differentiation status of cells, as well as the differentiation of stem cells into osteocytes and chondrocytes [56]. It is sometimes not possible to prepare a biomaterial with these desired properties using only one polymer. Therefore, composite or hybrid materials are created where a supportive scaffold could be added to comply with the necessary mechanical properties [57]. CS is used to create biomaterials for the regeneration of hard tissues such as bone and cartilage. In a hydrated state, CS scaffolds lack mechanical stability and therefore require extra modifications [58]. CS induces apatite deposition [59,60,61] and this phenomenon of the polymer has been used to enhance biomineralization of composite materials because CS favors calcium/phosphate ion accumulation and enhances the biomineralization potential of poly(ethylene glycol) diacrylate/CS-based hydrogel [52].

#### 3.2.1. Bone

CS mechanical properties are usually increased by the addition of hydroxyapatite due to its biological similarity to bone inorganic component [62]. In addition to hydroxyapatite, other composites, such as nano-zirconia/CS, nano-calcium zirconate/CS, and strontium-modified CS/montmorillonite composites with comparable mechanical properties were designed [63,64]. It was shown that MC3T3-E1 pre-osteoblastic cells when cultured on a CS-graft-polycaprolactone copolymer surface, in comparison to a tissue culture-treated polystyrene surface, show significantly higher alkaline phosphatase activity, deposition of calcium, and ECM synthesis [65]. For example, the addition of hydroxyapatite or bioglass to the matrix led to a compressive strength increase compared to CS alone. The polycationic nature of CS provides the possibility of designing polyelectrolyte complexes with polyanionic polymers to improve the mechanical properties of composite scaffolds [15,66]. In one study, CS/chondroitin/nano-bioglass-based polyelectrolyte composite material was developed with improved bioactivity, such as accumulation of apatite and increased expression of type-1 collagen by MG63 osteoblast-like cells in vitro and with osteointegration of the scaffold in vivo [67]. CS possesses active biomineralization properties and these could be further increased by introducing other polymers such as fucoidan [17,68] and bioglass [69].

Freeze-dried CS/gelatin scaffolds crosslinked with either glutaraldehyde or genipin support bone regeneration in vivo in mice inducing ECM production with minimal inflammatory reactions [70]. Thermosensitive hydrogel based on CS and beta-glycerophosphate was developed, however, it presented some biocompatibility issues due to an increased amount of substances required for gelation at body temperature. Recently, it was shown that the addition of TEMPO-oxidized cellulose nanofiber induced faster gelation and increased porosity with improved biocompatibility in vitro and in vivo in comparison to CS [71]. CS could be layered on top of metal (e.g., titanium) implants to increase osteointegration [72,73]. Composite materials based on polypyrrole/CS was synthesized through in situ electrochemical polymerization in oxalic acid medium and coated on 316L SS implants showing biocompatibility and protection against corrosion [74]. Recently, CS has been utilized in 3D printing for various tissue engineering applications [75]. CS-hydroxyapatite hydrogels were produced by a thermal cross-linking reaction using glycerol phosphate disodium salt and successfully printed on an extruder-based bioprinter. As a result, cells seeded on the printed scaffold increased osteogenic markers expression in comparison to 3D printed alginate and alginate-hydroxyapatite scaffolds [76].

#### 3.2.2. Cartilage

Regeneration of cartilage damaged by injury, disease (osteoarthritis), and degeneration as a result of aging is an important task in modern orthopedics. The approaches used to regenerate cartilage are microfracture, mosaicplasty, autologous chondrocyte, and biomaterial implantation [77]. An important limitation is the absence of blood vessels in the cartilage tissue, thus, the task of creating a biomaterial capable of stimulating the regeneration of cartilage under avascular conditions is the main goal of tissue engineering [78].

Designed biomaterials created for cartilage regeneration should be able to support cell proliferation and differentiation. Therefore, the use of cells and 3D scaffold together is a practical approach in tissue engineering [79,80]. The microstructural architecture, physicochemical, and biochemical properties of the scaffold should be able to provide a temporary template for cells and support ECM synthesis required for the formation of cartilage tissue [81]. This means that scaffolds, in addition to their biocompatibility and biodegradability, should be porous with interconnected pores [79]. Three-dimensional scaffolds, such as hydrogels, fibrous materials, and foams/sponges, are common scaffolds used in cartilage regeneration research [81]. Usually, scaffolds include cells (differentiated chondrocytes and stem cells) and bioactive molecules (peptides, growth factors, and cytokines). Hydrogels could offer high water content and support chondrogenesis potential, implantation without open surgery, and in situ scaffold formation. The low mechanical properties of hydrogels (*E* ≈ 200 kPa) [77] can be overcome with the use of solid supporters which improve the mechanical stability of the hydrogel [82].

CS as a natural material with a structural similarity to sulfated glycosaminoglycans provides a compatible microenvironment for chondrocyte proliferation, ECM synthesis, and chondrogenesis [78,80,83,84,85]. It was also demonstrated that chondrocytes cultured in CS-alginate beads reduce the expression of inflammatory cytokines (IL-6 and IL-8) and increase cartilage matrix components (hyaluronan and aggrecan) synthesis in vitro, in comparison to alginate beads alone [86]. CS derivative carboxymethyl-CS in a dose-dependent manner reduced the inflammatory profile of primary rat chondrocytes by reducing iNOS expression and upregulating the anti-inflammatory cytokine IL-10 in vitro [87]. In another study, the addition of hyaluronic acid-CS NPs to a pellet co-culture of the human infrapatellar fat pad (IPFP)-derived mesenchymal stem cells (MSCs) with osteoarthritic chondrocytes increased chondrogenic differentiation [88]. Human IPFP-MSCs seeded on 3D-printed CS scaffolds in chondrogenic media containing TGF-β3 and BMP-6 attach, proliferate, and differentiate into chondrocyte-like cells modulating the formation of cartilaginous tissue in vitro [89].

**Figure 2:**
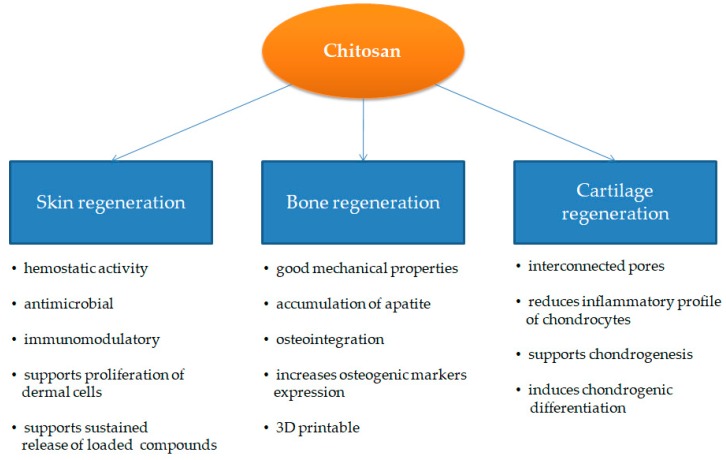
Main properties of chitosan (CS) used for skin, bone, and cartilage regeneration.

CS also interacts with collagen via electrostatic interactions between abundant amino groups and sulfo groups [90], and freeze-dried type 2 collagen-CS hybrid scaffold possesses improved stiffness in comparison to single component scaffolds, with a good porous structure resembling cartilage [91]. Moreover, type II collagen-CS scaffolds were also combined in the bi-layered scaffold with poly(lactic-co-glycolic acid) (PLGA) to further increase the mechanical and functional properties of biocomposites for cartilage regeneration [92]. CS-silk fibroin blends have also shown potential in cartilage regeneration [93,94]. One study found that bovine chondrocytes seeded on CS fibers made by an electrospinning method with a diameter of 300 nm have a 2-fold higher ratio of collagen II/collagen I in comparison to cells cultured on sponge-like scaffolds [95]. It is also important to note that a new type of supermacroporous scaffold made by a cryogelation method (cryogel) is gaining attention [13,14,15,16]. Supermacroporous (85–100 µm pore diameter) CS-agarose-gelatin scaffolds made by a cryogelation method (cryogel) possess good mechanical properties with an affable compression modulus of approximately 44 kPa of 5% cryogel at 15% deformation [96]. In vivo experiments for the repair of subchondral cartilage defects in female New Zealand white rabbits using CS-agarose-gelatin cryogels have shown the formation of hyaline cartilage without any hypertrophy markers by the fourth week post-implantation [97]. It is important to note that CS films induce human bone marrow MSCs to differentiate into chondrocyte-like spheroids in vitro via mTOR/S6K activation [98]. The main advantages of CS for skin, bone, and cartilage regeneration are highlighted in Figure 2.

### 3.3. Chitosan for Drug Delivery

As a natural component, CS presents itself as an interesting substance for drug delivery applications. It is biodegradable and susceptible to degradation by lysozyme produced by mucosal tissue [99] and chitinase produced by intestinal flora [100]. CS solubility increases under acidic conditions which is useful for oral delivery of the drug. However, low solubility under physiological pH possesses some limitations. Due to its mucoadhesive nature [101], CS has been used as a vehicle to deliver drugs to nasal [102], ocular [103], buccal [104], and pulmonary tissues [105]. For drug delivery purposes, CS is used in the form of nano/microparticles which is synthesized by emulsion, coacervation/precipitation, ionic gelation, reverse micellar methods, etc. [9]. The problem of solubility of CS under physiological conditions, which is required for efficient delivery of drugs, is usually solved by chemical modification of CS and includes quaternization, alkylation, acetylation, carboxymethylation, CS/polyol salt combinations, synthesis of *N*-trimethyl CS, generation of sugar-bearing CS, conjugation with polyethylene oxide, generation of glycol-CS, etc. [9,106]. For the encapsulation of hydrophobic substances, amphiphilic CS derivatives were synthesized [107]. CS moiety is modified with a long chain alkyl group with hydrophobic function, and the addition of hydrophilic groups, such as succinyl, to the amino group enables CS derivative to form micelles in aqueous media [108]. Micelle-forming *N*-succinyl-*N*′-octyl CS (SOC), *N*-octyl-*N*-trimethyl CS, and *N*-octyl-*O*-sulfate have been studied to deliver doxorubicin, hydroxycamptothecin (10-HCPT), and paclitaxel for tumor-targeted therapy with increased encapsulation [107].

CS NPs produced by an emulsion method is also used for the delivery of proteins and peptides [109]. Its high loading efficiency and sustained release of proteins in CS particles have been reported. However, it includes sequential cross-linking with tripolyphosphate, glutaraldehyde, and genipin, which could affect the biological activity of loaded proteins [110]. The emulsion method’s limitation could be prevented with the use of coacervation/precipitation, ionic gelation, polyelectrolyte formation, spray drying, and supercritical fluid drying methods [111]. CS microspheres loaded with recombinant human interleukin-2 have been prepared by a coacervation/precipitation method without the use of cross-linking agents [112]. The polycationic nature of CS is used to prepare polyelectrolyte complexes which spontaneously form upon mixing. For example, heparin is widely used with CS polyelectrolyte complex due to its ability to bind growth factors and cytokines [113,114,115,116,117]. In a recent study, CS-heparin NPs were used for the delivery of siRNA against vascular endothelial growth factor in human retinal epithelial cells (ARPE-19) with a 2-fold higher transfection efficiency in comparison to carrying plasmid DNA alone [118]. In addition to gene delivery, CS could be modified to deliver growth factors and cytokines [119]. The addition of a sulfate group to CS mimics heparin and heparan sulfate and retains its intrinsic antimicrobial properties [120]. Sulfated CS is able to bind fibroblast growth factor-2 [121] and bone morphogenetic protein-2 [122] and protects them from proteolytic cleavage [123]. Moreover, it was shown that sulfated CS binds to the proteins better than heparin [124].

CS as a non-viral gene delivery system has also been explored [9,125]. CS was used as a non-viral delivery system for plasmid transfection in 1995 [126]. The polycationic nature of CS interacts not only with negatively charged nucleic acid molecules forming a polyelectrolyte complex [127,128], but also with negatively charged cellular membranes, which results in increased uptake efficiency [129]. Nowadays, CS is used to deliver siRNA [130] and miRNA [131,132]. A widely used method for the preparation of CS for gene delivery is ionic gelation [133,134] and coacervation [135,136]. Recent application of CS and its derivatives for drug delivery is summarized in Table 1.

## 4. Conclusions

CS, as a natural polymer, is actively used in tissue engineering and regenerative medicine as a biomaterial alone, as well as in combination with other polymers. In addition to its suitable mechanical physico-chemical properties, CS has a natural ability to stimulate tissue regeneration. Active research is underway in improving CS-containing scaffolds for wound healing, bone, and cartilage regeneration. In addition to this, CS-containing polymers are being actively studied for the delivery of drugs for targeted tumor therapy and nucleic acid delivery in genetic engineering applications. Further research on the preparation of CS-containing scaffolds via 3D printing and cryogelation methods will facilitate the application of CS in biomedicine. CS, as a part of any material, could introduce valuable properties such as antimicrobial activity, mucoadhesiveness, and biocompatibility, which are in demand for biomedical use. We believe that further research on CS and the search for new variations in its use with other polymers will reveal even greater prospects and properties of this unique polymer in biomedical applications.

## Figures and Tables

**Table 1:** Chitosan and its derivatives for drug delivery. NPs: nanoparticles.

CS/Derivatives	Type/Delivery System	Application	Ref.
*N*-succinyl-*N*′-octyl chitosan (SOC)	Self-assembled polymeric micelles	Controlled anticancer drug release	[108,137]
		Tumor targeted therapy	[138,139,140]
		Biomedical optical imaging	[141]
*N*-octyl-*N*-trimethyl chitosan	Self-assembled polymeric micelles	Controlled anticancer drug release	[142]
		Tumor targeted therapy	[143]
*N*-octyl-*O*-sulfate chitosan	Self-assembled polymeric micelles	Absorption enhancement of anticancer drug	[144,145]
		Tumor targeted therapy	[146,147,148]
		Increasing stability of drug loaded liposomes	[149]
2-[phenylhydrazine (or hydrazine)-thiosemicarbazone]-chitosan	Powder	Pharmaceutical and food industries	[150]
(Ser-Ile-Lys-Val-Ala-Val) peptide-modified chitosan	Hydrogel	Skin substitutes for wound closure in mice	[53,151]
Galactosylated chitosan (GC)	NPs	Tumor targeted therapy	[152,153,154,155]
		siRNA delivery	[156,157]
*N*-palmitoyl chitosan (NPCS)	MPs and micelles	Tumor targeted therapy	[158,159]
*O*-palmitoyl chitosan (OPC)	Liposomes	Intestinal drug delivery	[160]
Hydroxyapatite/CS	NPs	Drug delivery	[161,162,163,164]
CS loaded with antioxidant NPs	Hydrogel	Drug release	[165]
PEGylated CS	NPs	Tumor targeted therapy	[166,167,168]
Chitosan-based vaccine	Polyelectrolyte, NPs	Intranasal CS-DNA vaccine	[169,170]
